# Targeting Intracellular Calcium Signaling ([Ca^2+^]_i_) to Overcome Acquired Multidrug Resistance of Cancer Cells: A Mini-Overview

**DOI:** 10.3390/cancers9050048

**Published:** 2017-05-09

**Authors:** Dietrich Büsselberg, Ana-Maria Florea

**Affiliations:** 1 Weill Cornell Medicine in Qatar, Qatar Foundation-Education City, POB 24144 Doha, Qatar; dib2015@qatar-med.cornell.edu; 2 Institute of Neuropathology, Heinrich-Heine University Düsseldorf, Moorenstraße 5, 40225 Düsseldorf, Germany

**Keywords:** chemotherapy, drug resistance, calcium signaling, cancer, epigenetic factors

## Abstract

Cancer is a main public health problem all over the world. It affects millions of humans no matter their age, gender, education, or social status. Although chemotherapy is the main strategy for the treatment of cancer, a major problem limiting its success is the intrinsic or acquired drug resistance. Therefore, cancer drug resistance is a major impediment in medical oncology resulting in a failure of a successful cancer treatment. This mini-overview focuses on the interdependent relationship between intracellular calcium ([Ca^2+^]_i_) signaling and multidrug resistance of cancer cells, acquired upon treatment of tumors with anticancer drugs. We propose that [Ca^2+^]_i_ signaling modulates gene expression of multidrug resistant (MDR) genes which in turn can be modulated by epigenetic factors which in turn leads to modified protein expression in drug resistant tumor cells. A precise knowledge of these mechanisms will help to develop new therapeutic strategies for drug resistant tumors and will improve current chemotherapy.

## 1. Cancer Is a Global Public Health Problem

Cancer is a main public health problem all over the world, affecting millions of people [1,2,3]. In this regard, the National Cancer institute (USA) states that “Cancer is increasingly a global health issue. In 2012, there were 14.1 million new cancer cases and 8.2 million cancer-related deaths worldwide. The World Health Organization projects that, by 2035, the world could see 24 million new cancer cases and 14.5 million cancer-related deaths a year” [4]. Furthermore, the World Health Organization warns that: “Global cancer rates could increase by 50% to 15 million by 2020” while “World Cancer Report provides clear evidence that action on smoking, diet and infections can prevent one third of cancers, another third can be cured” [5]. Therefore, to date, great attention and economical effort has been dedicated to discovering new strategies and methods of prevention and therapy of cancers worldwide.

## 2. Need of New Strategies to Overcome Drug Resistance

Although chemotherapy is the main strategy for the treatment of cancer, a major challenge limiting its success is the occurrence of an intrinsic or acquired drug resistance. Therefore, cancer drug resistance is a major impediment in medical oncology often resulting in a failure of the treatment. An acquired resistance to an anti-cancer drug or/and multiple drugs (which could be structurally and functionally different) could additionally result in cross-resistance to multiple drugs [6]. Clinically, drug resistance can exist prior to therapy or can be the consequence of cancer therapy [7] since Darwinian selection is also applicable in the clonal selection of tumor cells [8]. To resist chemotherapy, cancer cells might adopt different mechanisms e.g., modification in the metabolism and transport of chemotherapeutics, specific changes related to the chemotherapeutic targets as a result of mutations/amplifications as well as genetic redefinition that might lead to resistance to cell death upon chemotherapy [7]. Furthermore, tumor heterogeneity may also contribute to resistance; where small subpopulations of cells acquire or possess features that enable them to survive under selective pressure like the presence of a drug [7].

The activation of resistance pathways might in turn lead to multidrug resistance/cross-resistance, thus generating an even more challenging problem to overcome clinical diagnostics and treatment options [7]. Molecular research investigating the mechanisms of resistance utilizes molecular and biochemical approaches [6]. This mini-overview aligns with the significant efforts currently made all over the world to understand the mechanisms of cancer’s molecular pathology in order to improve the therapy of cancers. Thus, with our literature research, we aim to contribute and promote the development of effective anticancer strategies.

## 3. Hypothesis

**Figure 1:**
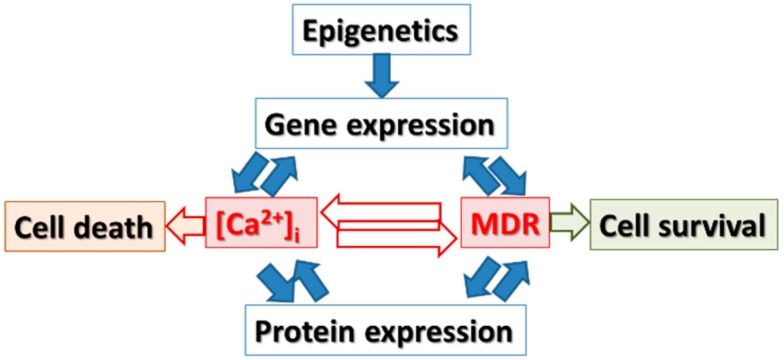
Hypothesis: the interconnections of the molecular mechanisms for the link between calcium signaling and multidrug resistance.

This mini-overview focuses on the interdependent relationship between intracellular calcium ([Ca^2+^]_i_) signaling and multidrug resistance of cancer cells, acquired upon treatment of tumors with anticancer drugs (Figure 1). We propose that [Ca^2+^]_i_ signaling modulates gene expression of multidrug resistant (MDR) genes which in turn can be modulated by epigenetic factors which consecutively leads to modified protein expression in drug resistant tumor cells. A precise knowledge of these mechanisms will help to develop new therapeutic strategies for drug resistant tumors and will improve current chemotherapy.

Under an epigenetic control, there are close relations connecting the intracellular calcium concentration ([Ca^2+^]_i_) to multiple drug resistance, changing cell death or survival as well as protein expression (Figure 1).

## 4. Gene Expression and Epigenetic Changes Are Important for Cancer Pathogenesis: Are They Targets to Overcome Drug Resistance of Anticancer Drugs?

Scientific evidence indicates that cancer is associated with modifications in gene expression due to mutations and epigenetic alterations. Genomic and epigenetic changes are important for cancer pathogenesis since molecular analysis of tumor specimens show distinct expression profiles depending on cellular or tissue types of carcinoma e.g., breast, lung, ovarian, prostate, neuroblastoma, glioblastoma. Furthermore, gene expression signatures have a strong prognostic value: e.g., gene signatures (i) correlate widely with the outcome for cancer patients; (ii) represent good predictors for the success of chemotherapy; and (iii) activate oncogenic pathways (for review see [9,10,11,12]). In addition, the expression of small non-coding RNAs, microRNAs (miRNAs), was linked to several human diseases, including cancer (for review: [11,13]). Thus, changes in mRNA and miRNA expression represent important targets for molecular therapy.

miRNA or cDNA microarrays studies—using different types of cancer specimens or cancer cell lines treated with anticancer drugs—are available in public databases such as: Cancer Genome Anatomy Project (http://www.ncbi.nlm.nih.gov/SAGE); Cancer Genome Atlas (TCGA; http://tcga-data.nci.nih.gov/tcga/); Stanford Microarray Database (http://smd.stanford.edu/); Gene Expression Omnibus (http://www.ncbi.nlm.nih.gov/geo/); Array Express (http://www.ebi.ac.uk/arrayexpress/); ONCOMINE, Cancer Profiling Database (http://www.oncomine.org/main/index.jsp); UNC-Chapel Hill Microarray (https://genome.unc.edu/); GOBO (http://co.bmc.lu.se/gobo/coexpressed_genes.pl); Oncogenomics (http://pob.abcc.ncifcrf.gov/cgi-bin/JK); Kaplan Meier Plotter Database (http://kmplot.com/analysis/index.php?p=background). The knowledge of previous studies is a powerful resource for more focused research as it enables the researcher to judge cancer risk, make a prognosis, and select the best therapy, thus offering the possibility to improve clinical applications. Overall, data mining in such databases helps to save resources that could be used for more meaningful experiments.

The treatment of cancers includes surgery, systemic treatment, and radiotherapy ([14]; reviewed in [15]). Usually, cancer treatments are based on stage, histology, and molecular biomarkers. Furthermore, the molecular prognostic and predictive biomarkers might represent helpful tools in selecting appropriate personalized anticancer therapies [10,11,12,16,17,18]. To date, the physician has a choice between multiple anticancer therapies; however, a positive response is often impaired due to acquired drug resistance.

Some examples of chemotherapeutic agents that are used for cancer treatment are platinum compounds (e.g., cisplatin (CDDP), carboplatin), temozolomide, doxorubicin, topotecan, paxitaxel, and irinotecan. Platinum-based drugs are used in a number of solid malignancies, including breast, testicular, ovarian, bladder, head and neck, esophageal, small and non-small cell lung, cervical, stomach, prostate, as well as Hodgkin’s and non-Hodgkin’s lymphomas, neuroblastoma, sarcomas, multiple myeloma, melanoma, and mesothelioma. In some clinical settings, CDDP represents the major therapeutic option but CDDP treatment often leads to chemo-resistance resulting in therapy failure [15,18,19,20,21,22].

**Figure 2:**
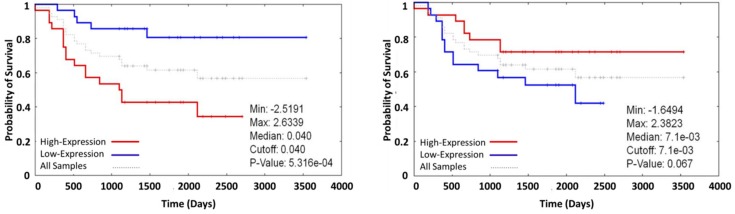
Overall survival of the neuroblastoma patients in correlation with the gene expression of ABC transporters. (**left**) ABCB1 (high expression = bad prognosis); (**right**) ABCC1 (low expression = bad prognosis) (taken from Oncogenomics database accessible under https://pob.abcc.ncifcrf.gov/cgi-bin/JK).

Mechanisms involved in acquired drug resistance of cancer cells are:

(i) a decreased intracellular drug concentration, e.g., due to the expression and activity of drug transporters (i.e., ATP-binding cassette (ABC) transporters) or metabolic enzymes (i.e., glutathione S-transferase, cytochrome P450 enzymes);
(ii) disturbances affecting the cell cycle arrest, apoptosis, and DNA repair (i.e., p53);
(iii) activation of signaling pathways related to progression of cancer;
(iv) epigenetic modifications (i.e., DNA methylation, miRNA, histone modification); and
(v) an increase or alteration in the availability of drug targets;
(vi) inactivation or compartmentalization of the agents;
(vii) inhibition of apoptosis and aberrant bioactive sphingolipid metabolism;
(viii) modification of gene expression

(reviewed in [19,23]; see also: [6,20,21,24]). In previous literature, it has been discussed that the limitation of the exposure to anticancer drugs and drug resistance in cancer cells is related mainly to the overexpression of ATP-binding cassette (ABC) efflux transporters: P-glycoprotein (P-gp/ABCB1), multidrug resistance-associated protein 1 (MRP1/ABCC1), and breast cancer resistance protein (BCRP/ABCG2) [25,26]. Thus, the occurrence of multidrug resistance as a result of modified gene expression during chemotherapy has serious implications (Figure 2) and might predict the outcome of the chemotherapy of cancer patients.

Cytotoxic drugs, such as doxorubicin, paclitaxel, or cisplatin damage the ‘genetic material’ of cells thereby interfering with proliferation of cancer cells. That might be caused by cell cycle arrest and/or apoptosis [18,19,20,21,23]. For instance, CDDP induces concentration-dependent cytotoxicity in diverse types of cancer cells due to targeting at the DNA level, but also with transcription and replication mechanisms. Furthermore, CDDP targets tumor cells due to its feature to trigger “on” signal transduction pathways (e.g., death receptor, calcium signaling, mitochondrial dependent apoptosis) that ends in tumor cell death [19,20,21]. Nevertheless, cancer cells might ultimately become CDDP-resistant due to modifications in the uptake mechanisms of the cells but also upon increased detoxification and drug efflux, increased DNA repair, as well as inhibition of apoptosis. To reduce CDDP resistance, combinatorial therapies by adding other drugs were developed, and are currently in use [20,21] but still might fail as the cancer cells could develop a multi-drug resistance (MDR).

Today, multidrug resistance, caused due to overexpression of ABC drug transporters, is a major problem in chemotherapy [24,25,26,27]. While, MDR and cancer invasiveness have been correlated, the molecular basis is not yet understood. The ABC transporter gene family expression was studied in different multidrug-resistant sub-lines including MCF7 cell line. When compared with their drug-sensitive parental lines, an overexpression of these genes in the multidrug-resistant cell lines was observed [28]. Furthermore, the expression of ABC transporters was directly associated with the survival of neuroblastoma patients [24].

Another key therapeutic challenge is to maintain a reduced tumor growth. After chemotherapy succeeds the loss of tumor cells frequently results in increased growth of drug resistant cells. In order to reduce the risk of ‘selecting’ resistant cells, specific strategies can be applied which depend on the type of chemotherapy used, the type of cancer cells, metastasis, the cell number associated with the tumor, as well as its growth rate; while the genetic and epigenetic variations might play a major role in the response to chemotherapy [8].

## 5. The Role of [Ca^2+^]_i_ Signaling in the Regulation of Expressing Genes which Are Relevant to Drug Resistance

Human tumors are diverse and respond in multiple ways to antitumor therapy [8,24,26]. Furthermore, variations in gene expression and regulation play an important role in the positive or negative outcome of an anti-cancer therapy [11,12,18,29]. For instance, gene expression profiling is essential to developing the breast cancer molecular classification and furthermore, biomarkers were identified which could predict outcome and response to chemotherapy, including drug resistance [10,30,31,32]. Thus, understanding the molecular mechanisms of gene expression and regulation in cancers is an initial step for the development of new—more efficient—therapies.

We propose that the regulation of gene expression in drug resistant cells via [Ca^2+^]_i_ plays an important role in acquired drug resistance but up to today, this hypothesis has not been investigated in detail. Modulations of [Ca^2+^]_i_ are ubiquitous cellular signals in tumor and non-tumor cells, keeping the balance between cell survival, growth, differentiation, and cell death. Changes in the intra- and extracellular Ca^2+^-concentration due to internal or environmental stimuli influence most cellular functions [32,33,34,35,36,37]. The maintenance of the tightly regulated [Ca^2+^]_i_ requires efficient regulating proteins (e.g., channels and ATPases). Calcium binding proteins sense and transform [Ca^2+^]_i_ signals to downstream cellular responses [33].

There are three major classes of membrane-associated proteins directly involved in Ca^2+^ regulation: (i) channels, (ii) ATP-ases (pumps), and (iii) exchangers. These proteins have different cellular and tissue distribution and their regulation occurs through multiple signaling pathways [31,32,36,37]. Altered expression of Ca^2+^-conducting channels and Ca^2+^-transporting pumps or calcium-binding proteins (such as S100 family, calpains, ORAI, TRPM, etc.) are characteristic for some cancers [31,32,33,35]. Recently, the review of [37] shows that the changes in the expression or activity of calcium channels and pumps are relevant for the pathology of cancers (patient samples and cell lines) for instance, in prostate cancer: TRPM8, TRPV6, ORAI1, TRPV2; breast cancer: TRPM8, TRPV6, TRPC6, PMCA1, PMCA2, SPCA2, ORAI1, ORAI3; lung cancer: IP3R2, CACNA2D2, TRPM8, PMCA, SERCA2; colon/colorectal: Cav. 1.1, Cav. 1.2, Cav. 3.1, Cav. 3.3, TRPM8, TRPV6, SERCA2, SERCA3, PMCA4; bladder: TRPV1; melanoma:TRPM1; oral: PMCA1, SERCA2; thyroid: TRPV6, SERCA2; gastric: IP3R3, Cav. 3.1, TRPC6; ovarian: TRPV6, TRPC3 (for review see [37]).

Thus, the ability of [Ca^2+^]_i_ to regulate both cell death and proliferation in addition with the potential of pharmacological modulation gives the clinicians a chance to use them as targets to improve cancer treatment [31,32,33,37], like the S100 family of calcium binding proteins, extracellular Ca^2+^-sensing receptor and calmodulin. The members of S100 family of proteins have EF-hand calcium binding sites that activate signaling pathways as well as Ca^2+^-sensors such as calmodulin and/or annexins. These calcium binding proteins are deregulated in cancer and can be used as diagnostic markers [33,38]. Furthermore, the extracellular Ca^2+^-sensing receptor (i) promotes differentiation in colon epithelial cells; (ii) is a tumor suppressor in colon cancer and breast cancer; (iii) promotes cellular sensitivity to cytotoxic drugs. Thus, drugs targeting the Ca^2+^-sensing receptor could be used as a therapeutic strategy [39,40]. Finally, calmodulin is involved in regulating cellular processes such as the balance of energy, inflammation, homeostasis of various physiological parameters (e.g., glucose, calcium), formation and development of blood cells, and cancer [41,42].

Scientific evidence points to the premise that [Ca^2+^]_i_ signaling affects cancer related processes:

- the cell motility, tumor invasion, and metastasis;
- angiogenesis;
- genotoxicity due to [Ca^2+^]_i_ modulation of the DNA damage response pathways thus controlling genomic stability and cell survival;
- transcription that takes place through [Ca^2+^]_i_ oscillation frequency, or transcription factor NFAT (nuclear factor of activated T cells);
- telomerase activity (e.g., S100A8 can inhibit the activity of telomerase contributing to cell immortalization);
- differentiation due to the capability of [Ca^2+^]_i_ signals to control the differentiation process through the extracellular Ca^2+^ sensing receptor and/or alterations in intracellular Ca^2+^;
- the cell cycle because [Ca^2+^]_i_ is a key regulator of the cell cycle and proliferation, through regulation of Ras activity or the subcellular localization of key proteins associated with tumorigenesis (e.g., Ca^2+^ controls PTEN nuclear localization);
- apoptosis due to:
  (a) Ca^2+^ accumulation in the mitochondria and activation of mitochondrial membrane permeabilization; while
  (b) the reduction in the Ca^2+^ content in ER is associated with resistance to apoptosis; or
  (c) Ca^2+^ homeostasis activates calpains, which are main regulators of apoptosis for cleaving pro- and anti-apoptotic proteins [31,32,34,36,37].

Furthermore, Frede and coworkers underlined that ion channels may be involved in multidrug resistance [43].

A possible mechanism was described by Akl and coworkers in 2013. The authors proposed that proto-oncogenes and tumor suppressors—which represent key players in cell survival, adaptation, and death—are in turn regulated by Ca^2+^-signals arising from the endoplasmic reticulum (ER). ER is found in close proximity to the mitochondria and therefore linked (e.g., Ca^2+^ transport mechanisms by inositol 1,4,5-trisphosphate receptor (IP3R) and the voltage-dependent anion channel (VDAC)). Thus, the amount of Ca^2+^ transfer from the ER to mitochondria might determine the susceptibility of cells to apoptotic stimuli and the apoptotic resistance of cells, thus resulting in the survival, growth, and proliferation of cells with oncogenic features [44].

Although extremely complex and heterogeneous, all cancer types share a few common traits: malignant cells are able to evade apoptosis; they have an uncontrolled cell growth followed by invasion and metastasis. All these cancer related processes might be controlled by second messengers such as Ca^2+^-signals; including the signaling pathways related to tumorigenesis and progression. Nevertheless, molecular mediators of cellular [Ca^2+^]_i_ homeostasis impact tumor dynamics and might result in deregulation of major oncogenes and tumor suppressors that are tightly associated with [Ca^2+^]_i_ signaling [45].

A model for capacitative calcium (Ca^2+^) entry proposing that depletion of endoplasmic reticulum stored Ca^2+^ levels leads to activation of plasma membrane calcium channels which mediate influx of Ca^2+^ from the extracellular space into cells was previously described, these biomolecules are known as store operated Ca^2+^ entry (SOCE). SOCE is vital to signaling pathways in health and disease while in cancer cells; SOCE plays an important role in cell cycle progression and proliferation, migration, metastasis, and evasion of apoptosis [46,47,48,49,50]. The Ca^2+^-selective store-operated current (ICRAC) is mediated by the endoplasmic reticulum (ER) Ca^2+^-sensor STIM1 and the store-operated Ca^2+^ (SOC) channel Orai1 but also the canonical transient receptor potential (TRPC) channel family, including TRPC1. In mammalian cells, several key components of SOCE have been described: STIM1-2, Orai1-3, and TRPC1-7; while in recent years, STIMs and ORAIs have been regarded as possible molecular targets for cancer diagnostic and therapeutics [47,49,50,51,52]. Anguita and Villalobo described in 2016 that there is a crosstalk between non-receptor Src-family kinases and the Ca^2+^ transient generated in activated cells by a variety of extracellular and intracellular stimuli that might result in stimulation of signaling pathways [53]. Nguyen et al. (2017) shows that two-pore channels, a class of NAADP- and PI(3,5)P2-sensitive Ca^2+^-permeable cation channels in the endolysosomal system of cells, are linked to cancer cell migration and might represent future targets to treat metastatic cancers [54].

Furthermore, microarray data from oncogenomics databases suggest that the expression of [Ca^2+^]_i_ related genes such as: RYR2, ITPR2, S100A4, S100A8, TRPV4, or TRPV6 is associated to the overall survival of neuroblastoma patients. Thus, the ability of Ca^2+^-signals to regulate pathways such as proliferation and apoptosis suggests that therapies that modulate Ca^2+^-signaling in cancer cells are a clinical alternative. Ca^2+^-channels and pumps which could have an altered expression in cancer and could be used as targets for chemotherapy [31,32]. Furthermore, the ability of calcium signaling to regulate gene expression, cell cycle, and apoptosis suggest that [Ca^2+^]_i_ homeostasis might have a crucial role in developing drug resistance during chemotherapy.

## 6. The Role of Epigenetic Changes in Gene Expression, Regulation of [Ca^2+^]_i_ Signaling and Drug Resistance Relevant Genes

In cancer, epigenetic modifications are widely studied. Most studies focused mainly on the changes in the DNA methylation pattern, which are stable and easy to measure, [55] followed by histone modifications and miRNA expression. The changes in the DNA methylation at specific CpG sites regulate cellular gene expression of specific genes the might be correlated with abnormal proliferation, apoptosis, migration, or invasion observed in cancer cells [56].

The global loss of genomic methylation—as well as regional hyper- and hypomethylation of genes involved in cell signaling, proliferation, and apoptosis—are thought to promote cell survival and tumor progression [57,58] and have potential clinical applications [59] since DNA methylation changes have been used to differentiate benign from malignant tissue and to predict tumor recurrence or patient outcome [55].

The downregulation of genes related to cancer might be the result of an epigenetic modification, thus, the development of drug resistance might also depend on epigenetic changes. DNA methylation occurs at cytosine nucleotides (CpGs) and is catalyzed by DNA methyltransferases. Areas of high CpG content (CpG islands) are found in mammalian promoters: the methylation state of CpG islands controls gene expression by blocking transcription [60,61]. Thus, DNA methylation might be involved in the expression of MDR genes [62] and calcium signaling related genes [63].

Accumulating evidence hints that specific changes in ion channel expression and function might be involved in cancer related processes and thus constitute important therapeutic targets and potential prognostic factors [56,59]. Thus, understanding how the deregulation of ion channels takes place specifically in cancer cells and the role of epigenetic modifications related to their over- or under-expression is an important task for cancer research. For instance, if the promoter of a gene that encoded an ion channel is hyper-methylated, it might have the outcome the loss in the mRNA expression followed by the loss of the encoded gene product while hypo-methylation is associated with increase of their expression. Furthermore, methylation status has been associated with a poor prognosis in cancer patients, thus with a potential of being used in diagnostic settings for predicting clinical outcome in cancer patients [56].

Day and Bianco-Miotto (2013) [55] discuss how the genome wide DNA methylation analysis discovered specific genes that have specific methylation pattern in malignant tissue or in recurrent tumors. These genes are potential biomarkers for diagnosis and prognosis such as the S100 calcium binding proteins, zinc finger transcription factors, homeobox genes, and potassium voltage-gated family members. Further genome wide DNA methylation profiles will define more specific and sensitive biomarkers for cancer diagnosis and prognosis but will also identify novel therapeutic targets that can be modulated using inhibitors of DNA methylation [55].

Increasing evidence shows that miRNAs are also involved in the biology of cancers. Aberrant miRNA expression occurs in different tumor types, thus demonstrating a causal role in tumorigenesis and indicating their use as biomarkers or therapeutic tools. Furthermore, miRNAs are involved in gene regulation through translational inhibition or mRNA decay. Thus, the expression of MDR genes and calcium signaling related genes could be regulated by miRNA. miRNA could potentially target any human mRNA as they are virtually involved in almost every biological process, including cell cycle regulation, cell growth, apoptosis, cell differentiation, and stress response. miRNAs modulate oncogenic or tumor suppressor pathways, while their expression can be regulated by oncogenes or tumor suppressor genes. Thus, these molecules could also represent a target to improve chemotherapy (reviewed in [13,23]).

Overall, epigenetic investigations could lead to the identification of additional targets linked to drug resistance that will allow the prediction and diagnosis of acquired MDR [58]. Certain DNA-methylation and histone de-acetylation inhibitors were recently approved by the US Food and Drug Administration as anti-cancer drugs [60].

## 7. Outlook

Cancer treatment is difficult due to the complexity and heterogeneity of each cancer type. Nevertheless, molecular characterization of cancers is getting more attention including the bioinformatic discovery of gene motifs that predict the response to anticancer drugs and the development of drug resistance [10,15,18,30,31] (see the Oncogenomics database). Among others, these gene signatures encode: (i) calcium signaling molecules, such as calcium channels and receptors, calcium binding proteins, calcium activated proteins; (ii) multidrug resistance proteins such as the ABC transporter family of proteins; (iii) apoptosis related genes; (iv) epigenetic regulators. Therefore, comprehensive and innovative studies are needed in order to obtain a better understanding of the role played by MDR genes, Ca^2+^-signaling components, and the epigenetic changes associated with such gene deregulations in drug resistance. The results might point to new possibilities to precisely target those molecules to avoid drug resistance.

Specific issues that have to be addressed in future research are:

ix. Identification and validation of genes that are deregulated upon chemotherapy: ((multi-) drug resistance and calcium modulating genes associated with cancer pathology (e.g., ABCB1, ABCC1, ABCG1, ITPR1, CACNG1, CACNA1D, CAMLG, CALB2, S100A, and TRPV family) (see recent work of [64]).
x. The establishment and characterization of the relationship between deregulation of calcium signaling and MDR expression upon chemotherapy in cancer cells.
xi. The identification and validation of selected MDR and calcium signaling related genes associated with acquired drug resistance of cancer cells.
xii. The verification on how and why specific manipulations of calcium signaling in drug resistant cancer cells will overcome the drug resistance.
xiii. To determine of the role of epigenetic manipulation of MDR and calcium signaling related genes will influence the acquired drug resistance of drug resistant cancer cells.
